# Using feminist methodologies to explore female genital mutilation/cutting and child marriage in low- and middle-income contexts

**DOI:** 10.3389/frma.2024.1330289

**Published:** 2024-12-18

**Authors:** Nicola Jones, Kate Pincock, Sarah Alheiwidi

**Affiliations:** ^1^Gender and Adolescence: Global Evidence (GAGE), ODI, London, United Kingdom; ^2^Refugee Studies Centre, Oxford Department of International Development, University of Oxford, Oxford, United Kingdom; ^3^Gender and Adolescence: Global Evidence (GAGE), Amman, Jordan

**Keywords:** FGM/C, child marriage, adolescent girls, qualitative methodologies, socio-ecological model, refugee, low- and middle-income context

## Abstract

This paper discusses how harmful practices such as child marriage and female genital mutilation/cutting (FGM/C) can be effectively explored through feminist methodologies that center the lived experiences of girls and young women affected by these issues. Eliminating harmful practices, which are rooted in gender inequality and have myriad life-course consequences for those who experience them, has become a global priority in recent years. However, dominant conceptualizations of the drivers and consequences of child marriage and FGM/C often fail to adequately engage with or reflect adolescent girls' own nuanced experiences and perceptions. A feminist epistemology underlines the need for research methods that can uncover and address dynamics of power and inequality, as well as the complexities of voice and agency surrounding these practices, both of which perpetuate harmful practices and marginalize the voices of those who are most affected. The paper discusses adaptations to two key sets of methodological tools used with adolescent girls in research on FGM/C in Ethiopia and on child marriage in Bangladesh, Ethiopia, Jordan, and Lebanon as part of the Gender and Adolescence: Global Evidence (GAGE) longitudinal study. The purpose of these tools was to address and shift power inequalities, both within the research process and in girls' lives more widely. The Social Network Hexagon, Marriage Chain, and Marriage Decision-Making Pairs tools were used to explore child marriage decision-making processes and dynamics and outcomes for adolescent girls. A vignette-based discussion on FGM/C and child marriage and a version of a Social Network Hexagon tool were used to improve understanding of adolescents' social networks and the extent to which these shape their options for change. We find that context-specific adaptations to the tools, determined through ongoing reflexive dialogue with local researchers, were key to creating space for deep reflection and engagement by girls. Exploring the wider structural and social dynamics that enable and constrain girls' agency and decision-making around harmful practices was also key. It enabled insights into the complex ways in which gender- and age-based social norms are exercised within girls' socio-ecological contexts, as well as possible entry points for change.

## 1 Introduction

Historically defined by the United Nations (UN) as “harmful traditional practices” to call attention to the cultural or socio-conventional motives that drive their continuation, child marriage and female genital mutilation/cutting (FGM/C) pose serious threats to the health, wellbeing, and rights of girls and women globally. The two practices are also often interlinked, with FGM/C often seen as a prerequisite for child marriage in contexts where both are practiced (Shell-Duncan et al., 2011; Boyden et al., 2013). Both are linked to social norms around sexual control of women and girls and the maintenance of traditions (Girls Not Brides, 2021).

Child marriage is a cultural and historical practice that is affected by interrelated economic and socio-political conditions as well as relational dynamics and gender norms (Jones et al., 2020; Emirie et al., 2021; Presler-Marshall et al., 2023). Research also highlights the role of poverty and material circumstances in driving child marriage (Kok et al., 2023), though in some contexts it may also be used by wealthier families in order to shore up family wealth (see Boyden et al., 2013). A significant body of research has observed the negative impacts of child marriage on girls' health and wellbeing (Gage, 2013; Malhotra and Elnakib, 2021; Siddiqi and Greene, 2022). Child marriage has been linked to early pregnancy and complications during childbirth, truncated education trajectories, limited labor market participation opportunities, and mental health impacts related to forced sexual relationships (Charles and Denman, 2013; Gage, 2013; McCleary-Sills et al., 2015; Kamal and Hassan, 2015; Efevbera et al., 2019). FGM/C can cause immediate and long-term pain, infection, sexual and reproductive health problems, and poor psychological wellbeing (World Health Organization, 2010).

However, dominant conceptualizations of the drivers and consequences of child marriage and FGM/C often fail to adequately engage with or reflect adolescent girls' own experiences and perceptions or properly situate these in relation to structural context (Freccero and Taylor, 2021; Lokot et al., 2021). A feminist approach to methodology directly engages with the power dynamics that marginalize certain voices and seeks to inform social change (Griffiths, 1995; Skeggs, 1997; Olesen, 2000). Consequently, feminist research methods with young women and girls have aimed to center their voices, elicit diverse viewpoints, and analyze power inequalities—both within the research process and in girls' lives more widely (Mitchell and Reid-Walsh, 2008). Postcolonial feminist theory has highlighted how categorization within geographic and historic matrices characterized by violence and domination results in the silencing of non-Western women and girls, and the homogenization of their experiences (Hooks, 1990; Mohanty, 2003; Chambers and Watkins, 2012). Postcolonial feminist work on methodologies has also emphasized the importance of continuous reflection by researchers upon their positionality, and the need to challenge assumptions and practices that can become reified through research itself (Mohanty et al., 1991; Lewis and Mills, 2003; Schurr and Segebart, 2012). This includes paying explicit attention to the broader structural factors in a given context that may shape girls' perspectives, opportunities, and decision-making.

This article reflects on how these principles informed the design and adaptation of certain research tools—mapping exercises, the “Marriage Chain,” “Marriage Decision-Making Pairs,” and adapted “Social Network Hexagon” tools—in research on FGM/C in Ethiopia and on child marriage in Bangladesh, Ethiopia, Jordan, and Lebanon as part of the Gender and Adolescence: Global Evidence (GAGE) longitudinal mixed-methods research program. The article describes how the tools were adapted in each context, and the opportunities created for deep reflection and engagement with the issues, both by adolescent girls and the main stakeholders in their socio-ecological environment. The article concludes with insights into how participatory methodologies informed by decolonial feminist theory can center adolescent girls and create in-roads for social change on complex and sensitive issues.

## 2 Background

### 2.1 Terminology and key concepts: harmful practices, child marriage, and FGM/C

Given the importance of discourse and language for constructing and interpreting the social world, it is important to first establish the semantic choices at the center of this article—not least because within feminism, contestation, and tensions over the terminology used to describe what have come to be known collectively as “harmful traditional practices” offer insights into some of the challenges involved in doing research on child marriage and FGM/C. The adoption of the shorter term “harmful practices” by the United Nations Convention on the Elimination of All Forms of Discrimination against Women (CEDAW) in 2014 (United Nations Office of the High Commissioner for Human Rights, 1979) was driven by feminist decolonial critiques that problematized the pitting of “modernity” against “tradition” that is implied in the discourse of “harmful traditional practices”, which situates women in the Global South as passive victims of violent patriarchy and individuals who lack personal agency (Mohanty, 1984; Spivak, 1988; Abu-Lughod, 2002). The language of “mutilation” in relation to the sociocultural practice of cutting of girls' genitalia has also been critiqued for the dualism it reproduces between the “natural” intact and secularized Western body, and the stigmatized, coerced, and oppressed bodies of women and girls in contexts where FGM/C is practiced (Njambi, 2005; Mahmood, 2010; le Roux and Bartelink, 2020). Similarly, policies and programs that have sought to address child marriage have been critiqued within some feminist postcolonial work as being vehicles for promoting neoliberal ideals around citizenship and social progress—that is, framing girls as conduits of economic development, rather than being concerned with girls' wellbeing, in the immediate and longer term (Khoja-Moolji, 2015).

In this article, the authors use the terms “harmful practices,” “child marriage,” and “FGM/C.” Although the latter term, in particular, has been contested, we argue that it reflects the need to engage with both the broad spectrum of degrees of physical impact and severity of FGM/C across contexts where fieldwork was conducted (including the different ages at which it is practiced and the implications for agency and consent) and avoids equivocation with “male circumcision”, which has far fewer risks and very different drivers.

### 2.2 Feminist research on child marriage and FGM/C

Feminist research seeks to inform efforts to address gender inequalities and improve the lives of women and girls and is thus critical to feminist activism (Jenkins et al., 2019). Feminist researchers adopt the perspective that research approaches have historically reflected the interests and values of white, heterosexual, and cisgender men, using traditional positivist social research methods that seek to uncover a singular and objective “truth” about a social issue (Sprague, 2016). Feminist research methodologies have highlighted how gendered power inequalities obscure and devalue women's voices within traditional research processes (Beetham and Demetriades, 2007). Postcolonial and decolonial feminist scholarship further calls attention to the embeddedness of gender relations within cultural, social, and economic practices, therefore requiring a nuanced understanding of local definitions of gender and of where real possibilities for transformation exist (Parpat and Parashar, 2019).

Using a postcolonial and decolonial feminist lens, a critical part of the research process is reflexivity about one's positionality in relation to research participants. Spivak (1988, 1999) uses the term “epistemic violence” to describe the silencing and disavowal of subjective experiences in order to constitute Western hegemonic explanations as the only true representation of reality. Analyses demand reflexivity on one's own consciousness and complicity with the structural conditions that legitimate particular explanations and understandings of situations and processes (Spivak, 1999). Moreover, Ahmed (2000) argues that researchers must recognize how encounters with participants are constituted within and may reproduce broader power inequalities and dynamics of exchange.

Historically, feminist research has not subjected age categories to the same degree of analysis as other social relationships. However, since the rise of childhood studies in the 1990s, there has begun to be more attention paid to the lives of girls—as opposed to women—as a group facing particular forms of marginalization, as well as being important social actors in their own right. Although similar in its emphasis on agency, early work within the rubric of “girlhood studies” critiqued childhood studies for paying inadequate attention to gender and interpreting childhood experiences in relation to boyhood (Harris, 2004). Mitchell (2016) also notes that prior to girlhood studies, there was little recognition of the feminist dilemma that exists in the relationship between adolescent girls and adult women, with the latter often implicated in harm against girls and younger women. Girlhood studies emphasizes the negotiation and construction of “girlhood” within particular moments and across global contexts; within the field, there has been growing attention to what constitutes ethical research practice when exploring the lives and experiences of girls in the Global South (Mandrona, 2016; Moletsane et al., 2021).

### 2.3 Adolescent girls' agency and decision-making

Though a body of research has explored the role of family dynamics and decision-making in the continuation or rejection of child marriage and FGM/C, there has been much less attention to adolescent girls' voice and agency in relation to these practices. This may be the result of a political framing of adolescent girls as victims of child marriage and FGM/C. Writing on child marriage, Bessa (2019) argues this narrative disregards the complexity of decision-making by girls and leaves them in a state of “informed powerlessness”, while Powell and Yussuf (2018) observe that the dominant narrative of FGM/C as gender-based violence can conflict with girls' understandings of the practice as part of traditions around gender identity, culture, and ethnicity in regions where it is common. Responding to this, a small but growing body of work has sought to explore girls' own perspectives and experiences of child marriage—and to a lesser extent FGM/C—through the lens of agency (on child marriage see, for example, Murphy-Graham and Leal, 2015; Kenny et al., 2019; Taylor et al., 2019; Baraka et al., 2022; on FGM/C see Palm et al., 2023). Critical analyses of the drivers of child marriage, in particular, have problematized the common assumption that child marriage is equal to forced marriage (with men and parents as the instigators of the practice), observing instead the agency of adolescent girls who choose to marry before the age of 18 (see also Jones et al., 2020; Emirie et al., 2021; Presler-Marshall et al., 2023).

This study has highlighted the need for a more nuanced understanding of consent and the role of parents in both practices. For example, Boyden et al. (2013) observe that concerns around increasing numbers of elopements by young people in Oromia, Ethiopia (where parents traditionally make arrangements for marriage), center on the risk to girls of abandonment and social exclusion should they defy their parents' wishes, as well as the detrimental impact on family honor of breaking with social conventions. This is echoed by Kenny et al. (2019) whose study of Somaliland and Puntland found that young people's increased agency contributed to more, not less, child marriage. Studies in India and Bangladesh have similarly found that girls may express a desire to marry young in line with parental preferences, which are themselves influenced by concerns about girls' safety and protection as well as by community norms (Abdul Azeez and Poonia, 2015; Hoque and Rashid, 2020). In conflict-affected contexts such as Syria, where insecurity and fears about safety and sexual violence are exacerbated, girls and their families may also see child marriage as an acceptable strategy for self-protection (Presler-Marshall et al., 2023).

Examining the nuances of how girls exercise agency in specific contexts, this literature explores how gendered social norms, poverty, the presence (or absence) of alternative opportunities, and other factors shape girls' decision-making. However, there remains a disconnect between this research and child marriage interventions, which Lokot et al. (2021) argue continue to be premised on an assumption that the practice is driven by a lack of knowledge about its harms and girls' rights; expanding girls' agency through raising awareness—it is presumed—will lead to girls resisting marriages forced on them by parents. The authors observe an aporia between critical academic understandings of the contradictory and contested nature of children's agency drawn from research with children themselves, and framings of agency within non-governmental organization (NGO) and UN agencies, noting that adopting a broader definition could improve interventions to address child marriage practices (Lokot et al., 2021).

A decolonial feminist lens that draws attention to structural context and interlocking inequalities offers a framework for engaging with child marriage and FGM/C in a more critical and reflexive manner. However, while postcolonial and decolonial feminist work (see, for example, Khoja-Moolji, 2015; Werunga et al., 2016) has drawn attention to the objectification and epistemic violence of assumptions about the passivity and victimization by patriarchy of women and girls in the Global South, adolescent girls' lived experiences have not been central to these analyses. Meanwhile, a currently substantial body of research within childhood studies has proposed a nuanced interpretation of adolescent girls' agency as relationally embedded and contingent (see, for example, Johnson-Hanks, 2005; Utas, 2005; Bell, 2007; Klocker, 2007; Murphy-Graham and Leal, 2015). These approaches are united in their emphasis on agency as relationally situated and dynamic; rather than being a quality possessed by an individual, it is better conceived of as a flow that shifts across time and in the context of different social networks and places. This approach aligns well with a postcolonial feminist lens and its attention to adolescent girls' lived experiences in relation to wider structural conditions—and the essential role of reflexivity in researching and interpreting findings.

### 2.4 Tools for researching child marriage and FGM/C

The challenge for feminist research on child marriage and FGM/C is to develop tools that allow a nuanced exploration of agency, context, relationality, and power dynamics. Within participatory research, the people affected by an issue are central to the process of generating data about it, with their participation and dialogue key to the process of social change (Breitbart, 2010). Critical and feminist work on participatory approaches has increasingly emphasized the need to address gendered and racialized power inequalities within methodology and praxis (Cornwall, 2000; McIntyre et al., 2004; Bradbury and Reason, 2005). Participatory methods are increasingly used with children and young people in part to actualize their rights enshrined in the United Nations Convention on the Rights of the Child (UNCRC) to have a say on matters that affect their lives (United Nations, 1989). Participatory approaches have also been used with adolescents to support activism around child marriage as part of development interventions designed to foster children and young people's agency to speak out about and challenge the practice; such approaches are recognized as a key strategy for the meaningful engagement of adolescents and communities (Plesons et al., 2021; Cuevas-Parra and Tisdall, 2022). However, one of the challenges for participatory research is translating findings into social change. Dialogue can spark critical consciousness about social norms and harmful practices, but it is essential that the process of research also engages with actors beyond those directly affected—those who hold positions of power and authority over children and young people.

In the following section, we discuss the research tools that were used in the GAGE study, focusing on how the principles of participatory research and decolonial feminist theory informed their design and adaptation.

## 3 Methods

### 3.1 Background to the GAGE research program

GAGE is a 10-year longitudinal study working in five low- and middle-income country (LMIC) contexts (Bangladesh, Ethiopia, Jordan, Lebanon, and Nepal), with a sample of 20,000 adolescent girls and boys aged 10–19 years at baseline (2018). This paper focuses on GAGE's qualitative and participatory workstreams in Bangladesh, Ethiopia, Jordan, and Lebanon, which involve a subset of ~1,000 adolescents (for more details, see Baird et al., 2021). This subset is organized into two age cohorts (10–12 and 15–17 years), with purposive sampling to ensure representation across the most marginalized adolescents, such as girls who have experienced child marriage, adolescents with disabilities, and refugee adolescents. GAGE adopts a mixed-methods approach to exploring adolescents' experiences throughout the second decade of life in six key domains: health; psychosocial wellbeing; economic empowerment; education; voice and agency; and bodily integrity and freedom from age- and gender-based violence. Child marriage and FGM/C are included within this latter domain.

Child marriage is prevalent in many of the communities with which GAGE works, particularly in Ethiopia, which is ranked fourth globally in terms of the absolute burden of child marriage, and in Bangladesh, which is ranked second (Girls Not Brides, 2023). Among the communities GAGE works with that are affected by forced displacement—Rohingya families in Bangladesh, and Palestinian and Syrian refugees in Jordan and Lebanon—child marriage rates are also high. In Jordan, GAGE conducted research with Syrians and Palestinians living in the capital, Amman, as well as Syrians living in Azraq and Zaatari refugee camps and informal tented settlements in rural areas, and Palestinians in Jerash refugee camp. In Lebanon, research included Palestinian and Syrian refugees in urban and rural Baalbek, and Palestinian refugees (including “double” refugees from Syria) in Wavel and Ein el-Hilweh camps. Studies have estimated that among Syrian families, rates of child marriage have increased 4-fold since the start of the crisis in 2013 (United Nations Population Fund, 2017; El Arab and Sagbakken, 2019). Although the rate of child marriage among Palestinian refugees in Jordan and Lebanon has been declining, it is widely recognized to be an issue driven by norms about girls' mobility and family honor as well as material deprivation (Hamad et al., 2021). In Bangladesh, this article draws on GAGE research with Rohingya refugees in Cox's Bazar, where approximately 40–50% of girls who have fled Myanmar are married. The practice has been escalating during their displacement due to reduced monitoring and regulation of marriage unions (Guglielmi et al., 2021).

In Ethiopia, child marriage and FGM/C are practiced to varying degrees in different parts of the country. There has been a sharp decline nationally in rates of child marriage in the past two decades, but this masks huge regional variance, with different complex drivers across contexts that are essential to unpack (Jones et al., 2020). The research discussed in this article was undertaken in East Hararghe (Oromia region) and in several sites within Afar and Somali regions. The most recent large-scale quantitative data also show that more than 65% of girls and women aged 15–49 years have experienced FGM/C [Central Statistical Agency (CSA), Ethiopia and ICF, 2017; UNICEF, 2020]. Afar and Somali regions have the highest rates of FGM/C in the country (91 and 98%, respectively); in Oromia, the government estimated the rate at ~76% in 2016, but more recent GAGE research found rates in East Hararghe to be closer to 89% [Central Statistical Agency (CSA), Ethiopia and ICF, 2017; Presler-Marshall et al., 2022]. As noted elsewhere in literature, the drivers of FGM/C vary, as do its physical invasiveness and consequences (Boyden et al., 2013; Presler-Marshall et al., 2022). The WHO outlines four categories of FGM/C: type 1 (clitoridectomy), type 2 (excision), type 3 (infibulation), and type 4 (other). Type 3 is considered to be more severe and associated with increased health risks, followed by type 2, but risks can vary based on the type and amount of tissue that is cut and other factors (World Health Organization, 2008). Based on WHO categorizations, in Oromia region, where FGM/C is typically undertaken in early infancy though can be undertaken later in puberty, type 1 is the norm, whereas in Somali and Afar regions, type 3 is typical (World Health Organization, 2010).

### 3.2 GAGE research tools for exploring child marriage and FGM/C

The qualitative and participatory research tools described below were used in both group and paired settings with multiple girls or pairs of girls, in dyads with parents or grandparents, and with individual girls (for a full description of each tool and how to use it, see Jones et al., 2019). In all settings, consistently listening to and centering the voices and experiences of adolescent girls and creating space for them to lead the focus of discussions and activities is crucial to addressing power relations between researcher and participant, in line with participatory approaches and values. Adolescents meet regularly with the same researchers over time, allowing them to build up relationships of trust and understanding. Written informed consent for participation in this study was provided by the participants' legal guardians/next of kin for participants under the age of 18 years, except in communities with low literacy levels where written consent was not appropriate. In these communities, the information from the written consent forms was provided orally, and consent sought verbally and then confirmed again with the respondent at the beginning of the interview recording. Consistent with a commitment to a feminist ethic of care by the research team (see Cockburn, 2005), robust referral processes were also a core component of the research protocol. GAGE has a detailed referrals protocol where we link respondents based on need to specialist service providers. Researchers can draw on these to assist adolescents who have experienced child marriage, gender-based violence or FGM/C and would like to access services such as healthcare or psychosocial support, integrated support services, and/or legal mechanisms.

GAGE works closely with partner research institutes in Lebanon, Ethiopia, and Bangladesh, which facilitate access to local communities. GAGE research is undertaken by experienced researchers of the same gender as participants and with shared linguistic and cultural backgrounds. Although this is helpful for establishing rapport, this latter commonality is particularly important for understanding the relational nature of agency. It also allows connections to be made between insights into the social, economic and political conditions at particular points and decision-making within communities about child marriage and FGM/C (Murphy-Graham and Leal, 2015; Hoque and Rashid, 2020; Lokot et al., 2021; Presler-Marshall et al., 2023). Researchers' knowledge of local conditions and norms can also enable a clearer understanding of regional and other differences that necessitate changes to the core research protocols and questions for each country. Through collective debriefings, researchers reflect on their interpretations of situations and experiences and discuss how the wider structural environment affects individual participants' experiences. This is done though exchanges of views and experiences with other researchers that are chaired and facilitated by a senior researcher. It is in these spaces that adaptations to tools were also proposed, discussed, reviewed and agreed. Research findings were later shared through national and regional dissemination workshops and through infographics translated into local languages.

#### 3.2.1 Child marriage

To explore opportunities for and constraints to girls' agency around child marriage, GAGE employed a set of complementary tools that were adapted and added to in each national and subnational context. To begin with, in each community, researchers undertook a gender norms community mapping exercise. This study aimed to engage community leaders and service providers (including clan and religious leaders, health extension workers, school teachers, women's and youth association community liaison officers, and district-level officials from bureaus of health, education, justice, and social development) in a discussion about the patterning of social and gender norms affecting adolescents, how they have shifted over time, and the underlying reasons for change, stasis, and/or backlash. Community leaders and service providers were identified through the researchers' knowledge of the local context and the profiles of different types of actors, including who was influential. They were recruited to the study through outreach by researchers and snowballing on the basis of suggestions from community members and local leaders. To understand evolving community norms around child marriage, researchers included probing questions covering awareness of existing or new laws, program interventions, possible economic drivers, uptake of education for girls, and the presence of local champions for change.

A second tool, “Marriage Decision-Making Pairs”, was used to provide insights into the parallel decision-making logics of the key actors (such as parents or other relatives) in girls' families, and those of their husband and in-laws (in the case of married girls). In this case, we identified outliers through a snowballing process of parents and recently wed husbands who have either supported child marriage (i.e., allowed/endorsed a marriage to a child bride) or parents who have opposed it (i.e., actively resisted pressures around a child marriage). Those outliers were then interviewed in pairs (one “conservative”; one “progressive”) to jointly reflect on vignettes that tell the story of two characters with opposing views about child marriage decision-making and the role of parents, in-laws, and grooms.

A third tool, the “Social Network Hexagon”, asked girls to identify who they are close to within their family, school, neighborhood/community, work environment (if relevant), friendship network and online (if relevant), and who they would turn to for advice and support (and why) within each sphere and overall, when navigating pressures around child marriage. This tool was used with both ever-married and unmarried girls, to explore differences in their relational support networks and the implications for their marriage trajectories.

The fourth tool, the “Marriage Chain”, was used with ever-married girls (including those who are separated, divorced, or widowed) to explore, without judgment, the decision-making processes, and constellation of actors that shaped the events leading up to their marriage. The tool helped unpack the factors that influenced the timing and type of a girl's marriage, her experiences and degree of agency during the process, as well as in decision-making once she is married, and possible exit options (including separation and divorce). The tool allowed a married or divorced girl to reflect on the factors that led to her marriage, their relative importance, the relative power exercised by different stakeholders for and against her decision, and at what junctures and to what extent she was able to exercise voice and agency.

#### 3.2.2 Female genital mutilation/cutting

In order to explore adolescent perceptions around and experiences of FGM/C in Ethiopia, GAGE researchers used a similar combination of tools, beginning with a gender norms mapping exercise with community leaders and service providers. This aimed to understand the patterning of norms and practices around FGM/C in the community and the factors that have shaped any changes related to acceptance of the practice, the type of FGM/C carried out, by whom, and at what age.

Informed by the very different community norms and practices that the mapping exercise elucidated, the interviews with adolescent girls drew on two complementary tools. The first was a vignette, which explores the experiences of two characters with divergent approaches to hearing about the risks of FGM/C, including legal, psycho-emotional, and health risks. The tool is used with girls in a group; the researcher reads the vignette aloud and invites reflections and comments from participants, with the conversation led by girls, often among themselves. One of the girls in the story dismisses this new information about risks, reasoning that FGM/C is critical to religious and cultural identity and any challenges associated with it need to be borne for these reasons, while a second girl finds the information about risks of the practice compelling, wants to avoid undergoing FGM/C, and is frustrated by the lack of support she finds in her family and community to help her resist these pressures.

The second tool used during interviews with girls was the “Social Network Hexagon”, which discusses six spheres of support that adolescent girls might be able to draw on when facing challenges, including resisting FGM/C. In the same way that the tool was used to explore child marriage, it asked girls to identify who they are close to within their family, school, neighborhood/community, work environment (if relevant), friendship network and online (if relevant), and who they would turn to for advice and support (and why) within each sphere and overall, to tackle pressures related to FGM/C.

## 4 Results

Informed by a postcolonial feminist ontology and a recognition of the contextual and relational nature of agency, the core methodological toolkits were adapted for use in the four countries to create space for girls' authentic voices and perspectives to be articulated in ways that accommodate, rather than simplify the complexities of their lives. Table 1 provides an overview of the core research questions about child marriage and the questions that were added or altered by local research teams in each country. Table 2 provides the same information for the research on FGM/C in three regions of Ethiopia (Afar, Oromia, and Somali). These overviews are followed with a discussion about the process of the adaptations and their consequences for how evidence about FGM/C and child marriage was generated in line with a participatory and feminist research agenda.

**Table 1:** Research questions on child marriage and adaptations per country.

**Tool**	**Participants**	**Core questions**	**Bangladesh adaptations**	**Ethiopia adaptations**	**Jordan adaptations**	**Lebanon adaptations**
Community gender norms mapping	Community leaders and service providers (religious leaders, clan leaders, local government officials, including from health, education, justice, women and children's affairs/social development, other literate adults)	In what ways have norms and practices toward age and type of marriage changed over time and why?	To what extent and in what ways has forced displacement shaped marriage practices among the Rohingya community?	To what extent and in what ways have awareness-raising efforts related to the law shaped marriage practices? What about changes in education and economic opportunities (or lack thereof)? How has conflict affected girls' risks of child marriage?	To what extent and in what ways has forced displacement shaped marriage practices among the Syrian refugee community?To what extent and in what ways do truncated rights to education and employment among Palestinian refugees in Jordan affect girls' risks of child marriage?	The same as Jordan but with additional questions, including: “How has the economic and governance crisis in the country since 2019 affected the drivers of child marriage?”
Marriage Decision-Making Pairs	Father, mother and husband of married girls; father and mother of unmarried girls.	To what extent and in what ways do laws and legal sanctions, awareness-raising programs, social assistance programs to support education, role models, and media campaigns shape parental decision-making around time and type of marriage for their children?	What are your experiences of insecurity in the Rohingya community? What is the role of local leaders (*majhi*)? (with probes about their role in sanctioning marriages and shaping attitudes and also access to support from humanitarian actors around reporting of child marriage risks)	What is the role of religious leaders and of brokers? What is the role of clan leaders in shaping marriage practices?	Same as core questions, with additions: Did you experience pressures to remarry, and if so, from whom? (based on rebuilding family networks after conflict-related deaths). How did you respond and why?	As per Jordan, with additional questions, including: What additional pressures have you experienced following the economic crisis and how have these shaped the drivers of child marriage, if at all? What has been the impact on communities of forced deportation of young men back to Syria, including on pressures to marry (e.g., as a protective measure)?
Social Network Hexagon	Adolescent girls (in pairs or individually)	Who can you rely on for support if you or someone close to you is at risk of child marriage? What sort of support would you seek from them and why?	Same as core questions, with additions: To what extent could you rely on the support from local leaders (*majhi*)? What about the support of humanitarian actors in the camps? How would this support differ? Who would you prioritize turning to and why?	Same as core questions, with additions: To what extent could you rely on support from religious leaders or clan leaders? Why?	Same as core questions, with additions: To what extent could you rely on support from local leaders (*mukhtar*) or religious leaders? What about local governorate officials? What about humanitarian actors, e.g., UN Refugee Agency (UNHCR), United Nations Relief and Works Agency for Palestine Refugees in the Near East (UNRWA), NGOs (international and local)? Among these actors, who would you first turn to and why?	Same as core questions, with additions: To what extent could you rely on support from local leaders (*mukhtar*) or religious leaders? What about humanitarian actors, e.g., UNHCR, UNRWA, NGOs (international and local)? Among these actors, who would you first turn to and why?
Marriage Chain	Adolescent girls who are ever-married (i.e., currently married, separated or divorced or widowed) (individually)	What factors at the family (economic and psycho-emotional), peer, community, legal, service actor, and programmatic levels (as relevant) shaped your decisions at each stage in the marriage decision-making process? What were the risks if you had resisted? What support could you rely on and from whom?	Same as core questions, with additions: What support, if any, could you access in the camp if you had wanted? Why/why not?	Same as core questions, with additions: Could you rely on support—and, if so, what forms of support—from any actors, including local officials, girls' club leaders, school teachers and principals, health extension workers, and program implementers? Did you encounter pressures from any actors, e.g., clan leaders (Afar), elders, peers at adolescent-only traditional dances (*shegoye* in East Hararghe and *sadah* in Afar) to marry?	Same as core questions, with additions: What support, if any, could you access to mitigate pressures to marry? For example, from humanitarian actors? From community center teachers? From the courts/legal system?	Same as core questions, with additions: What support, if any, could you access to mitigate pressures to marry? For example, from humanitarian actors? From community center teachers? From the courts/legal system?

**Table 2:** Research questions on FGM/C in three regions of Ethiopia.

**Tool**	**Participants**	**Core questions**	**Afar region adaptations**	**East Hararghe (Oromia region) adaptations**	**Somali region adaptations**
Community gender norms mapping	Community leaders and service providers (religious leaders, clan leaders, local government officials, including from health, education, justice, women and children's affairs/social development, and other literate adults)	How have norms and practices related to FGM/C changed over time and what factors (economic, education, legal, awareness-raising, media, health, etc.) have contributed to change or stasis?	Have there been any changes in the type of the practice (e.g., from more invasive type 3 to less invasive forms), the age at which it is carried out, and by whom (e.g., traditional birth attendant, cutter, health worker, doctor, mother/grandmother)? (The federal law has not been ratified at the regional state level so less relevant.) What role do cultural and religious factors play in perpetuating the practice? How important is this compared to awareness of health risks?	Have there been any changes in the type of the practice (e.g., from more invasive type 2 to less invasive forms), the age at which it is carried out, and by whom (e.g., traditional birth attendant, cutter, mother/grandmother)? To what extent is the practice being abandoned, and if so, by whom? What role is the law playing in this? How important is this compared to awareness of health risks? Religious imperatives? Cultural reasons?	Have there been any changes in the type of the practice (e.g., from more invasive type 3 to less invasive forms), the age at which it is carried out, and by whom (e.g., traditional birth attendant, cutter, health worker, doctor, mother/grandmother)? To what extent, if at all, has the practice become medicalized? (The federal law has not been ratified at the regional state level so less relevant.) What role do cultural and religious factors play in perpetuating the practice? How important is this compared to awareness of health risks?
Vignettes	Adolescent girls, adolescent boys, fathers, mothers (in focus group discussions)	To what extent and in what ways do laws and legal sanctions, awareness-raising programs, role models and media campaigns shape parental decision-making around whether to practice FGM/C and, if so, at what age and what type, and by whom?	The same questions; however, for questions related to type of FGM/C and age at which it takes place, there is little scope for agency of adolescent girls as the practice is done in infancy or early to mid-childhood, and therefore questions would explore norms and decision-making by parents and other stakeholders, including: What do you think about the decisions of the girls and parents in the vignette story? Does this resonate with the dominant norms in your community? Why/why not? What about if this were a sibling or a future child of yours—would your actions be similar or different? Why/why not?	For questions related to agency, in some communities the practice is done in early to mid-adolescence so questions were added to explore girls' relative agency in FGM/C decision-making: What influenced the decision for the person in the vignette to undergo FGM/C? Why did she make that choice? Does this resonate with your experience? Why/why not?	As in East Hararghe, in some communities the practice is done in early to mid-adolescence so questions were added: What influenced the decision for the person in the vignette to undergo FGM/C? Why did she make that choice? Does this resonate with your experience? Why/why not?
Social Network Hexagon	Adolescent girls (in pairs or individually)	Who can you rely on for support if you or someone close to you is at risk of FGM/C? What sort of support would you seek from them, and why?	What actors might offer support to girls at risk of FGM/C (e.g., health extension workers, teachers, NGO staff, local officials, relatives with higher education/in urban areas)?	Same as core questions, with additions: is the role of girls'/gender club leaders at school in supporting girls at risk from FGM/C?	What actors might offer support to girls at risk of FGM/C (e.g., health extension workers, teachers, NGO staff, local officials, relatives with higher education/in urban areas)?

### 4.1 Mapping exercise for child marriage

The mapping exercise with local community leaders and service providers elicited insights into the structural barriers and prevailing gender norms that shape girls' ability to exercise agency. The tool was, in turn, used to inform probing questions in interviews with parents and girls, which revealed how decision-making about marriage is changing over time in line with wider structural shifts, within communities and nationally/internationally. This reflects feminist decolonial work that draws attention to the relationship between gender norms and socioeconomic factors—the dynamism of which is often overlooked in research that positions harmful practices as static and unchanging “traditions”.

In East Hararghe (Oromia region, Ethiopia), for example, community stakeholders emphasized that norms around marriage had shifted over the past two decades away from parent-arranged marriages to adolescent-initiated marriages, due to several factors. As one girl explained, a booming *khat* (a stimulant plant native to eastern Africa) cash crop economy has had a demotivating effect on adolescent girls' education—a protective factor against child marriage—as adolescents can generate a better income from selling *khat* than from public sector jobs that require higher education. Another emerging trend that was cited as contributing to marriages starting in early adolescence was the rise of local brokers who are contracted by prospective grooms to put pressure on adolescent girls to accept offers of marriage.

In the case of Syrian refugee families in Jordan, community leaders noted the limited labor market opportunities for refugees in host communities and the absence of conducive conditions to return to Syria. They explained how this meant that for many parents, secondary education is not affordable and even when it is, there are high perceived risks of sexual harassment and a potential loss of honor for girls traveling to school. As such, child marriage is seen as offering some level of economic security for their daughters in the absence of other options to invest in their futures.

It should also be noted that social norms, investigated through the mapping exercise, not only do not discourage early marriage, but often encourage it. This is especially because early marriages are associated with larger family size and the opportunity to rebuild families and clans devastated by conflict.

### 4.2 Marriage decision-making pairs

The Marriage Decision-Making Pairs tool provided rich insights into the motivations of parents of sons and daughters, and of young men, regarding child marriage, the degree of autonomy afforded to adolescent girls as prospective brides, as well as the types of information, pressures, sanctions, and incentives it would take to shift their stance at different junctures in the decision-making process—i.e., from pre-engagement through to formal ceremony. The tool often thus reveals moments and situations where power relations are reproduced—or even disrupted—and what this means for understanding girls' agency.

These findings on parental decision-making logics were, in turn, used to inform and shape the questions researchers asked in the interviews with adolescent girls themselves in each community. During daily debriefings, insights from this tool were shared among team members, facilitating triangulation of information about local gender norms on child marriage in real time during data collection. These debriefings were also a key space for reflexivity about interpreting emerging findings, with researchers encouraged to consider how broader power dynamics—especially those connected to age and gender inequalities—may shape their perspective on the data that was being generated by the tools.

This tool was important for revealing the relationality of agency and the range of actors involved in decision-making about marriage, which can be obscured within analyses that center on girls' individual capacities. In the case of parents and husbands in Ethiopia's Afar region, where *absuma* marriage (i.e., to a maternal cousin) dominates, it was revealed that while the timing of marriage could be delayed, there was limited scope to permit choice as to a different marriage partner if there were no cousins of marriageable age. In many cases, parents as well as prospective grooms therefore faced very strong pressure from other clan members to accept a marriage offer. This meant that the social cost of resisting a marriage offer and allowing a daughter more agency could be very high; it was thus relatively rare.

In host communities in Bangladesh, parents focused on the perceived limited return to girls' education as a driver for child marriage. After marriage and childbearing, girls are expected to stay at home and do caring and domestic work. So while in the GAGE sample in Bangladesh, there are relatively few girls married in early adolescence, continuing education beyond grade 10 is rare and marriage in late adolescence is accepted, especially in rural communities, with limited scope for girls to pursue alternative pathways outside marriage.

### 4.3 Marriage chain

While much of the global literature and many program interventions focus on family-arranged marriages, the Marriage Chain tool enabled us to elicit and explore a much wider diversity of marriage types than is typically recognized. This is partly due to the purposive sampling and adaptation of research questions to reflect local realities, informed by the detailed knowledge of the local research teams about the key issues affecting marriage decisions. Types of marriage included “arranged marriages” that were sprung on girls with very little advance notice (for example, in Rohingya communities), and “arranged marriages” that were compelled due to pregnancy—either consensual or as a result of sexual assault (for example, in Amhara, Ethiopia). Girls also described “arranged marriages” that girls acquiesced to but in an environment of constrained choices (for example, in Amhara, Ethiopia, and in Bangladesh). There were also descriptions of “cousin marriages” arranged by girls' uncles (Palestinian refugees, Syrian refugees in Jordan and Lebanon). In Afar, Ethiopia, “*absuma*” marriages were often decided soon after birth or in early childhood, endorsed by the clan. Finally, girls described adolescent-initiated marriages decided on by adolescents only (Oromia, Ethiopia) or with broker involvement (also in Oromia).

Challenging reductive and problematic narratives that depict girls who married as children as inherently lacking in agency, the tool elicited responses that showed a complex range of psycho-emotional, familial, social (both peer and broader community pressures), and economic reasons underpinning their decision-making logic leading up to their marriage. For some girls, escaping a challenging natal family environment characterized by poverty, hunger, or violence was a key driver; for others, maintaining a good relationship with family members and not wanting to burden their parents or to shame them socially (by being deemed unmarriageable if they left marriage “too late”) was an important consideration. Others still were strongly influenced by pressure from peers and/or the perceived opportunity to secure community respectability and voice through marriage. As shown in Table 1, by asking about these wider dynamics, the tool also helped reveal that even in contexts where girls were opposed to marriage, they still sought to exercise agency around choice of partner, timing of the marriage, and whether they were able to continue in education post-marriage.

### 4.4 Mapping exercise for female genital mutilation/cutting

As shown in Table 2, the gender norms mapping exercise used with key stakeholders such as community leaders, social workers, and medical professionals enabled insights into regional differences around the drivers and practice of FGM/C across Ethiopia. Although this is already recognized to some extent within extant literature, probing changes to the practice over time (as with the tool on child marriage) enabled researchers to understand better what is effective for promoting social change, and conversely what factors contribute to continuities in regions where FGM/C remains widespread. The non-judgmental and open-ended nature of questioning was also able to elicit more detailed responses from participants than is often possible in discussions about such a sensitive subject, especially in the context of legal reforms and targeted initiatives that often use stigmatizing and alienating language to describe the practice.

In the Amhara region, where FGM/C is typically undertaken in early infancy and is type 1, community stakeholders emphasized that the practice was increasingly being abandoned as a result of exposure to awareness-raising activities in schools, and with local communities more aware of the legal ban and the negative health effects of FGM/C. Given this increased awareness, as shown in Table 2, researchers asked questions about who girls could go to for support around FGM/C.

By contrast, in the Somali region, where the practice is typically carried out in middle childhood and early adolescence prior to marriage and tends to be type 3 (infibulation), community stakeholders revealed that the practice is still very commonplace but is evolving toward either the use of fewer stitches (with some minimal improvements in health outcomes for girls and women) or, increasingly, reliance on medical practitioners to carry it out, using anesthesia to reduce pain and medical complications, especially hemorrhaging. As shown in Table 2, questions around norms and practices related to FGM/C were adapted for the Somali region to more specifically ask about changes in the type of practice and to ask about whether the practice has become more medicalized.

### 4.5 Vignettes and Social Network Hexagon tools for FGM/C

The vignettes and Social Network Hexagon were participatory tools that sought to put girls in control of related discussions. Creating space for dialogue to be led by girls enabled the researcher to indirectly explore a girl's perceptions of the practice, motivations for the practice among her family, peers, and community, and access to information and support should a girl seek not to undergo FGM/C. There is also space within the tool for girls to disclose their own experiences or that of friends or relatives if they are comfortable to do so. Key to the success of this tool was the trusting relationships that researchers had established with participants over time. The Social Network Hexagon was used one-on-one, and entailed a more detailed discussion with girls about their sources of support and advice, and the influence those individuals had on girls' decision-making and wellbeing.

One issue that emerged strongly during these interviews was that in communities where the practice is very widespread, even if an individual girl or family wants to abandon the practice, the social pressures to go ahead—given that FGM/C is closely linked to notions of honor and marriageability—are very substantial; resisting can therefore have very high social costs. While girls often noted that they have close relationships with their mothers, aunts, and female friends, in the case of FGM/C, these were often the actors who were most committed to continuing the practice. By contrast, in communities where there are a larger number of girls and women who have not been cut and where norms around marriageability are shifting, and especially where there are local role models and champions (such as health extension workers or girls' club teachers), girls have far greater opportunities to resist the practice.

## 5 Discussion

A key characteristic of the tools presented here was the space that they created for adolescent girls to express themselves on their own terms and in authentic ways. Countering the notion that girls who married as children are passive victims, the Marriage Decision-Making Pairs tool enables girls to tell their own stories, and describe the multiple decisions that led to marriage—albeit within a very constrained set of choices. These choices are also exercised in the context of the threat of stigma, social ostracization, violence, limited future options for marriage, and limited awareness about accessing support to opt out prior to marriage. As the questions in Table 1 show, the starting point of the Marriage Chain tool is to understand how adolescent girls felt at each stage leading up to marriage and afterward; it does not start with any assumptions about whether girls did or did not want to be married. As such, it creates space for girls to direct the trajectory and focus of discussions and thus allows for powerful insights around the varied and interconnected sources of girls' agency—and constraints—within the marriage decision-making process.

Using non-directive questions also allows for girls to choose whether to disclose information—or not. In Jordan, the Marriage Pairs tool was used both in individual interviews, to elicit specific stories and complexities, and in group interviews, to better understand local community norms and better understand similarities and differences in dynamics around child marriage decision-making, and avenues for support and services, across host and refugee communities. By being invited to reflect on an imaginary peer in the FGM/C vignette tool, girls in the Ethiopian research were able to place distance between themselves and the subject matter; they did not have to reveal their own experiences, unless they wanted to. This can also help avoid distress or embarrassment that can be associated with the disclosure of personal experience (Bradbury-Jones et al., 2014), while also discussing an issue that is often of critical importance in a girl's life—shaping her current and future health and wellbeing as well as social opportunities and social status within the community.

Importantly, a decolonial and feminist perspective emphasizes that young people's agency and participation must be understood as embedded within a network of intergenerational power relationships as well as situated within systems of cultures and values that are the site of contestation and power struggles (Kurtiş and Adams, 2017; Liebel, 2020). Recognizing the interdependencies of relationships and the role of children and adolescents as active social agents is fundamental to feminist care ethics (Cockburn, 2005). The tools described in this article offer opportunities for insights into these interpersonal dynamics and the factors that influence parent and caregiver perceptions. The mapping exercises used for this research engage with community leaders and service providers but can also be used with family members, husbands, and girls themselves; the Marriage Decision-Making Pairs and Marriage Chain tools are designed to be used with girls and their parents and husbands. Recognizing the interrelated nature of child marriage, in particular with contextual factors, the tools also provide space for discussions about the broader socio-political and economic issues that are factored into decision-making about child marriage.

The methods described here are only as effective as the researchers who are using them; the success of the tools in exploring adolescent girls' agency is thus dependent on researchers' reflexivity, curiosity, listening, and probing skills, as well as an ability to interpret data according to local contexts (Pincock and Jones, 2020). The longitudinal nature of the GAGE study allows for research tools to be adapted over time to improve the quality of the data generated. However, it also supports relationships of care, trust, and understanding to be established between participants and researchers, as these require time to cement, and for complexities in girls' own understandings, attitudes, and behaviors to be explored and unpacked. The longitudinal structure of the study, and the relationships that are established therein, in turn, support adaptations to research tools, because researchers are only able to be responsive to the lived realities of participants if they have a compassionate understanding of their experiences.

Feminist research forefronts the interpersonal and subjective condition of research interactions, and the need for researchers to reflect on the implications of their own age, gender, race, sexuality, nationality, and other social identities for their interactions, lines of questioning, and interpretation of data (Simandan, 2019; Potts et al., 2022). Within postcolonial feminist approaches, the relationship between researchers and participants is also recognized as an important site for the contestation of unequal power relations through processes of care, compassion, and accountability (Spivak, 2000; Sander, 2021). As noted, the GAGE researchers using the tools discussed in this article are local researchers who have a deep understanding of the issues facing young participants and have built up connections with them over time. Through debriefings during fieldwork, researchers have a space to reflect on their positionality and their understanding of emerging findings; they are also supported to engage critically with the data as it is generated and reflect on opportunities for subsequent adaptations to the tools.

The tools used in the context of GAGE research explicitly sought to center adolescent girls' experiences and shift power inequalities, both within research interactions and in their lives more broadly, with the aim of feeding into social change processes where girls' priorities and needs are center stages. This is a key tenet of feminist research methods (Beetham and Demetriades, 2007). A vital component of initiating social change was the collective and intergenerational dialogues that the tools enabled, and the impact of these on participants' perspectives. Vignette-based discussions within a group can also provide the opportunity for participants to engage in critical discussion about a topic, and this can itself support conscientization that is central to social norms change (Pincock et al., 2023). A number of girls (and indeed parents) who participated in the Marriage Decision-Making Pairs interviews pointed out that if they had had a chance to discuss their situation using the tool prior to marriage, they would not have proceeded with the marriage—suggesting that the tool could also be employed explicitly for purposes of awareness-raising, and social and behavioral change.

The interview-based tools (Marriage Chain, Marriage Decision-Making Pairs, vignettes, and Social Network Hexagon) were also designed to be adapted and added to by researchers to the age, interests, and capacities of participants and the context in which they live, allowing for young people's expression and agency within the research process and aligning with participatory objectives around voice and addressing power relations. This flexibility is particularly important for researching potentially sensitive issues such as FGM/C and child marriage. The vignettes offered a means for free reflection by participants on issues that were recognizable and relevant to their lives; the abstract format of encouraging reflection on the experiences of characters in a story enabled adolescents to make their own decision about how much they wanted to reveal about their own views. This was particularly helpful for younger adolescents, for whom discussing issues such as marriage or FGM/C might be more embarrassing or difficult due to age-related norms that foreclose such discussions, especially with adults (Pincock and Jones, 2020). Vignettes are also consistent with a participatory and feminist agenda in that they are concerned with the personal meanings that young participants attribute to issues, and center on dialogue, rather than assessing knowledge or practices specifically (Palaiologou, 2017). By identifying sources of support for girls, the Social Network Hexagon also offered insights into entry points for social change on harmful practices.

This approach to knowledge production is a key part of the broader strategic agenda of the GAGE program to understand “what works” to address harmful practices and expand girls' capabilities. Although changes in individual perspectives are an important element of social change, it is also essential that feminist research addresses the structural drivers of continuities in harmful practices such as FGM/C and child marriage. By working through local research teams, engaging with community stakeholders throughout the research process, and amplifying girls' voices locally and nationally (in publications and public engagement efforts), GAGE research can have an impact on policy and programming specifically because of its nuanced and inclusive approach to understanding harmful practices.

Finally, it is important to acknowledge some of the limitations of the research tools used, especially in relation to the actualization of a feminist research agenda and participatory objectives. Although guided by a participatory agenda, the design of the tools was not always participatory in that the basic format used and certain elements (for example, the content of the vignettes) were decided on in advance of their use, though researchers always acted on adolescents' feedback on the structure or content, and drew on formative qualitative research to inform the development of the tools. Possibilities for social change and the advancement of justice for girls are also constrained by issues such as resourcing within countries for accountability mechanisms and support systems, but more importantly, by entrenched inequality, poverty, and protracted displacement. As our evidence shows, these factors are integral to the continuation of child marriage and FGM/C. Another limitation to note is the uneven capacities and willingness of community leaders and service providers to engage in a reflective discussion on these practices, especially for those recently in post, given the high turnover of such roles in some settings. Where interviews yielded limited insights, we sought where possible to select someone else in an analogous role by snowballing with other engaged key informants.

## 6 Conclusion

Overall, our findings highlight the value of feminist research methodologies that are both contextually grounded and provide non-judgmental spaces for adolescent girls to reflect on opportunities and constraints for agency and voice related to practices such as child marriage and FGM/C. Approaches that forefront these objectives are critical to understanding the diversity and complex interplay of gender norms and structural economic, legal, and social factors that shape opportunities for change over time. Given that girls' agency and voice are powerfully shaped by their micro/family and peer networks as well as the broader community environments in which they live, it is vital to twin tools that allow girls to construct their own narratives with exercises that map the evolving and dynamic nature of social and gender norms over time, as well as the complex pressures and opportunity structures that parents and men and boys also face. This methodological toolkit demonstrates that these practices are neither static nor immutable, but instead require interventions that are contextually tailored and factor in girls' varied but constrained sets of choices and decision-making entry points within complex socio-ecological environments.

Moreover, we find that context-specific adaptations to the tools—by experienced and reflexive researchers with shared linguistic and cultural backgrounds, who have built a relationship with participants over time—were key to creating space for deep reflection and engagement by girls. Exploring the wider structural and social dynamics that both enable and constrain girls' agency and decision-making around harmful practices was also key. Reflecting the emphasis within postcolonial feminist methodologies as to a critical interrogation of how structural dynamics shape interpersonal experiences, this approach enabled insights into the complex ways in which gender- and age-based social norms and inequalities are exercised within girls' socio-ecological contexts—as well as identification of possible entry points for change.

## Data Availability

The raw data supporting the conclusions of this article will be made available by the authors, without undue reservation.
